# Supplementary material for: Development and validation of a minimum requirements checklist for snakebite envenoming treatment in the Brazilian Amazonia
Source: PLoS Negl Trop Dis. 2024 Jan 19;18(1):e0011921. doi: 10.1371/journal.pntd.0011921 (PMC10829989; doi:10.1371/journal.pntd.0011921)
Supplement: S1 File — (DOCX) [file pntd.0011921.s001.docx]

**PART 1 – UNIT INFORMATION**

Carefully read the checklist of Health Facilities. Then fill in the fields below.

Part 1: below each page of the form there is a field for comments and suggestions to be made.

| Site data | | | | | |
| --- | --- | --- | --- | --- | --- |
| Municipality: |  | | Verification date: |  | |
| Unit name: |  | | | | |
| Unit ID: | | National Register of Health Establishments (CNES): | | | |
| Address: | |  | | | |
| Geographic coordinates: | |  | | | |
| Unit management: | | ( ) Federal ( ) State ( ) Municipal ( ) Military | | | |
| Open during week days: | | ( ) 4 hours ( ) 8 hours ( ) 12 hours ( ) 24 hours | | | |
| Open during weekend: | | ( ) Yes ( ) No  If yes,  ( ) 4 hours ( ) 8 hours ( ) 12 hours ( ) 24 hours | | | |
| Electricity: | | ( ) Network ( ) Generator ( ) Solar power | | | |
| Back up electricity (in case of power outage): | | ( ) No ( ) Generator ( ) Reusable ice pack/styrofoam box | | | |
| Contact is by: | | ( ) No reliable communication ( ) Land line ( ) Cell phone ( ) Internet ( ) Radio | | | |
| Is the unit part of the national immunization plan network? | | ( ) Yes ( ) No | | | |
| If yes, what does it offer: | | ( ) Vaccines ( ) Rabies injection ( ) Antivenom | | | |
| Driving distance from nearest hospital (km): | |  | | | |
| Time (hours) to reach the hospital: | |  | | | |
| Transport mode for patient transfer: | | ( ) By river ( ) Terrestrial ( ) Air  Multiple options possible | | | |
| Transport possible during: | | ( ) 8 hours ( ) 12 hours ( ) 24 hours | | | |
| Person responsible for the information | | | | | |
| Name: | | | Role: | | Signature: |
|  | | |  | |  |

Other Suggestions:

**PART 2 – HUMAN RESOURCES**

For each item there will be four evaluation options, according to chart 1.

Table 1. Possible valuations of the item in accordance with the judge's decision.

| Evaluation | Definition |
| --- | --- |
| ESSENTIAL | The presence of the item is mandatory for the storage of the antivenom and administration to the patient. |
| UNESSENTIAL | The presence of the item is indifferent to the storage of the antivenom and administration to the patient. |
| DESIRABLE | The presence of the item is not mandatory, but it increases the quality of storage and administration of the antivenom to the patient, offering greater convenience/comfort for health professionals and patients. |
| UNDESIRABLE | The presence of the item may be detrimental to the storage of the antivenom and administration to the patient. |

Each item must be validated 3 times, according to the type of unit, according to quadro 2.

Table 2. Classification of health facilities according to the ability to care for patients who are victims of snakebite envenoming.

| Type of Unit | Definition |
| --- | --- |
| Type 1 | Unit that is capable of providing care to patients, including antivenom treatment, but refers all patients to a higher-level unit after this procedure. |
| Type 2 | A unit that is able to provide care to patients, including antivenom treatment, but refers critically ill patients to a higher-level unit after this procedure. |
| Type 3 | Hospitals; units capable of caring for patients, including antivenom treatment for all children of serious two patients. |

| **No. of Item** | **Description** | **The evaluation of the expert as to the need for the item with the type of unit** | | |
| --- | --- | --- | --- | --- |
|  |  | **Type 1** | **Type 2** | **Type 3** |
| 2.01 | Does the unit have a registered nursing technician/nursing assistant? | ( ) Essential  ( ) Unessential  ( ) Desirable  ( ) Undesirable | ( ) Essential  ( ) Unessential  ( ) Desirable  ( ) Undesirable | ( ) Essential  ( ) Unessential  ( ) Desirable  ( ) Undesirable |
| 2.02 | If yes, how many during in each shift? |  | | |
| 2.03 | Did the technicians/assistants have specific training in the snakebite treatment protocol (classes, workshops, etc) during or after their formation? | ( ) Essential  ( ) Unessential  ( ) Desirable  ( ) Undesirable | ( ) Essential  ( ) Unessential  ( ) Desirable  ( ) Undesirable | ( ) Essential  ( ) Unessential  ( ) Desirable  ( ) Undesirable |
| 2.04 | If yes, how many during in each shift? |  | | |
| 2.05 | Describe the availability (desired) of these professionals: | ( ) 4 hours ( ) 8 hours ( ) 12 hours ( ) 24 hours | | |
| 2.06 | Does the unit have a nurse? | ( ) Essential  ( ) Unessential  ( ) Desirable  ( ) Undesirable | ( ) Essential  ( ) Unessential  ( ) Desirable  ( ) Undesirable | ( ) Essential  ( ) Unessential  ( ) Desirable  ( ) Undesirable |
| 2.07 | If yes, how many during in each shift? |  | | |
| 2.08 | Did the nurses have specific training in the snakebite treatment protocol (classes, workshops, etc) during or after their formation? | ( ) Essential  ( ) Unessential  ( ) Desirable  ( ) Undesirable | ( ) Essential  ( ) Unessential  ( ) Desirable  ( ) Undesirable | ( ) Essential  ( ) Unessential  ( ) Desirable  ( ) Undesirable |
| 2.09 | If yes, how many during in each shift? |  | | |
| 2.10 | Describe the availability (desired) of these professionals: | ( ) 4 hours ( ) 8 hours ( ) 12 hours ( ) 24 hours | | |
| 2.11 | Does the unit have a physician? | ( ) Essential  ( ) Unessential  ( ) Desirable  ( ) Undesirable | ( ) Essential  ( ) Unessential  ( ) Desirable  ( ) Undesirable | ( ) Essential  ( ) Unessential  ( ) Desirable  ( ) Undesirable |
| 2.12 | If yes, how many during in each shift? |  | | |
| 2.13 | The physicians had specific training in the snakebite envenoming treatment protocol (classes, workshops, etc.) during or after its formation? | ( ) Essential  ( ) Unessential  ( ) Desirable  ( ) Undesirable | ( ) Essential  ( ) Unessential  ( ) Desirable  ( ) Undesirable | ( ) Essential  ( ) Unessential  ( ) Desirable  ( ) Undesirable |
| 2.14 | If yes, how many during in each shift? |  | | |
| 2.15 | Describe the availability (desired) of these professionals: | ( ) 4 hours ( ) 8 hours ( ) 12 hours ( ) 24 hours | | |
| 2.16 | Does the unit have a clinical analysis laboratory? | ( ) Essential  ( ) Unessential  ( ) Desirable  ( ) Undesirable | ( ) Essential  ( ) Unessential  ( ) Desirable  ( ) Undesirable | ( ) Essential  ( ) Unessential  ( ) Desirable  ( ) Undesirable |
| 2.17 | If yes, how many laboratory technicians during each shift? |  | | |
| 2.18 | Describe the availability (desired) of these professionals: | ( ) 4 hours ( ) 8 hours ( ) 12 hours ( ) 24 hours | | |
| 2.19 | Does the unit have a pharmacist? | ( ) Essential  ( ) Unessential  ( ) Desirable  ( ) Undesirable | ( ) Essential  ( ) Unessential  ( ) Desirable  ( ) Undesirable | ( ) Essential  ( ) Unessential  ( ) Desirable  ( ) Undesirable |
| 2.20 | If yes, how many during in each shift? |  | | |
| 2.21 | Did pharmacists have specific training in the snakebite envenoming treatment protocol (classes, workshops, etc.) during or after their training? | ( ) Essential  ( ) Unessential  ( ) Desirable  ( ) Undesirable | ( ) Essential  ( ) Unessential  ( ) Desirable  ( ) Undesirable | ( ) Essential  ( ) Unessential  ( ) Desirable  ( ) Undesirable |
| 2.22 | If yes, how many during in each shift? |  | | |
| 2.23 | Describe the availability (desired) of these professionals: | ( ) 4 hours ( ) 8 hours ( ) 12 hours ( ) 24 hours | | |

**PART 3 - EQUIPMENT**

| **No. of Item** | **Description** | **The evaluation of the expert as to the need for the item with the type of unit** | | | **Role / Use** |
| --- | --- | --- | --- | --- | --- |
|  |  | **Type 1** | **Type 2** | **Type 3** |  |
| 3.01 | Pulse oximeter | ( ) Essential  ( ) Unessential  ( ) Desirable  ( ) Undesirable | ( ) Essential  ( ) Unessential  ( ) Desirable  ( ) Undesirable | ( ) Essential  ( ) Unessential  ( ) Desirable  ( ) Undesirable | Evaluation of vital signs |
| 3.02 | Clinical thermometer | ( ) Essential  ( ) Unessential  ( ) Desirable  ( ) Undesirable | ( ) Essential  ( ) Unessential  ( ) Desirable  ( ) Undesirable | ( ) Essential  ( ) Unessential  ( ) Desirable  ( ) Undesirable |  |
| 3.03 | Sphygmomanometer | ( ) Essential  ( ) Unessential  ( ) Desirable  ( ) Undesirable | ( ) Essential  ( ) Unessential  ( ) Desirable  ( ) Undesirable | ( ) Essential  ( ) Unessential  ( ) Desirable  ( ) Undesirable |  |
| 3.04 | Bag-valve-mask (BVM) | ( ) Essential  ( ) Unessential  ( ) Desirable  ( ) Undesirable | ( ) Essential  ( ) Unessential  ( ) Desirable  ( ) Undesirable | ( ) Essential  ( ) Unessential  ( ) Desirable  ( ) Undesirable | Emergency care |
| 3.05 | Intubation Kit for children | ( ) Essential  ( ) Unessential  ( ) Desirable  ( ) Undesirable | ( ) Essential  ( ) Unessential  ( ) Desirable  ( ) Undesirable | ( ) Essential  ( ) Unessential  ( ) Desirable  ( ) Undesirable |  |
| 3.06 | Intubation Kit for adults | ( ) Essential  ( ) Unessential  ( ) Desirable  ( ) Undesirable | ( ) Essential  ( ) Unessential  ( ) Desirable  ( ) Undesirable | ( ) Essential  ( ) Unessential  ( ) Desirable  ( ) Undesirable |  |
| 3.07 | Stretcher | ( ) Essential  ( ) Unessential  ( ) Desirable  ( ) Undesirable | ( ) Essential  ( ) Unessential  ( ) Desirable  ( ) Undesirable | ( ) Essential  ( ) Unessential  ( ) Desirable  ( ) Undesirable |  |
| 3.08 | Stretcher trolley | ( ) Essential  ( ) Unessential  ( ) Desirable  ( ) Undesirable | ( ) Essential  ( ) Unessential  ( ) Desirable  ( ) Undesirable | ( ) Essential  ( ) Unessential  ( ) Desirable  ( ) Undesirable | Administration of antiophidic serum and other medicines |
| 3.09 | Hospital armchair | ( ) Essential  ( ) Unessential  ( ) Desirable  ( ) Undesirable | ( ) Essential  ( ) Unessential  ( ) Desirable  ( ) Undesirable | ( ) Essential  ( ) Unessential  ( ) Desirable  ( ) Undesirable |  |
| 3.10 | Intravenous infusion pole | ( ) Essential  ( ) Unessential  ( ) Desirable  ( ) Undesirable | ( ) Essential  ( ) Unessential  ( ) Desirable  ( ) Undesirable | ( ) Essential  ( ) Unessential  ( ) Desirable  ( ) Undesirable |  |
| 3.11 | Phlebotomy armrest | ( ) Essential  ( ) Unessential  ( ) Desirable  ( ) Undesirable | ( ) Essential  ( ) Unessential  ( ) Desirable  ( ) Undesirable | ( ) Essential  ( ) Unessential  ( ) Desirable  ( ) Undesirable |  |
| 3.12 | Defibrillator | ( ) Essential  ( ) Unessential  ( ) Desirable  ( ) Undesirable | ( ) Essential  ( ) Unessential  ( ) Desirable  ( ) Undesirable | ( ) Essential  ( ) Unessential  ( ) Desirable  ( ) Undesirable | Emergency care |
| 3.13 | Pharmacy /Vaccine Refrigerator‎ | ( ) Essential  ( ) Unessential  ( ) Desirable  ( ) Undesirable | ( ) Essential  ( ) Unessential  ( ) Desirable  ( ) Undesirable | ( ) Essential  ( ) Unessential  ( ) Desirable  ( ) Undesirable | Storage of antiophidic serum |
| 3.14 | Domestic type refrigerator | ( ) Essential  ( ) Unessential  ( ) Desirable  ( ) Undesirable | ( ) Essential  ( ) Unessential  ( ) Desirable  ( ) Undesirable | ( ) Essential  ( ) Unessential  ( ) Desirable  ( ) Undesirable |  |
| 3.15 | Refrigerator thermometer | ( ) Essential  ( ) Unessential  ( ) Desirable  ( ) Undesirable | ( ) Essential  ( ) Unessential  ( ) Desirable  ( ) Undesirable | ( ) Essential  ( ) Unessential  ( ) Desirable  ( ) Undesirable |  |
| 3.16 | Oxygen cylinder | ( ) Essential  ( ) Unessential  ( ) Desirable  ( ) Undesirable | ( ) Essential  ( ) Unessential  ( ) Desirable  ( ) Undesirable | ( ) Essential  ( ) Unessential  ( ) Desirable  ( ) Undesirable | Emergency care |
| 3.17 | Oxygen flowmeter | ( ) Essential  ( ) Unessential  ( ) Desirable  ( ) Undesirable | ( ) Essential  ( ) Unessential  ( ) Desirable  ( ) Undesirable | ( ) Essential  ( ) Unessential  ( ) Desirable  ( ) Undesirable |  |
| 3.18 | Water bath | ( ) Essential  ( ) Unessential  ( ) Desirable  ( ) Undesirable | ( ) Essential  ( ) Unessential  ( ) Desirable  ( ) Undesirable | ( ) Essential  ( ) Unessential  ( ) Desirable  ( ) Undesirable | Blood clotting test |
| 3.19 | Glass tubes | ( ) Essential  ( ) Unessential  ( ) Desirable  ( ) Undesirable | ( ) Essential  ( ) Unessential  ( ) Desirable  ( ) Undesirable | ( ) Essential  ( ) Unessential  ( ) Desirable  ( ) Undesirable |  |
| 3.20 | Emergency trolley/cart crash | ( ) Essential  ( ) Unessential  ( ) Desirable  ( ) Undesirable | ( ) Essential  ( ) Unessential  ( ) Desirable  ( ) Undesirable | ( ) Essential  ( ) Unessential  ( ) Desirable  ( ) Undesirable | Emergency care |
| 3.21 | Stethoscope | ( ) Essential  ( ) Unessential  ( ) Desirable  ( ) Undesirable | ( ) Essential  ( ) Unessential  ( ) Desirable  ( ) Undesirable | ( ) Essential  ( ) Unessential  ( ) Desirable  ( ) Undesirable | Evaluation of vital signs |
| 3.22 | Ice pack | ( ) Essential  ( ) Unessential  ( ) Desirable  ( ) Undesirable | ( ) Essential  ( ) Unessential  ( ) Desirable  ( ) Undesirable | ( ) Essential  ( ) Unessential  ( ) Desirable  ( ) Undesirable | Storage and transportation of antiophidic serum |
| 3.23 | Styrofoam box | ( ) Essential  ( ) Unessential  ( ) Desirable  ( ) Undesirable | ( ) Essential  ( ) Unessential  ( ) Desirable  ( ) Undesirable | ( ) Essential  ( ) Unessential  ( ) Desirable  ( ) Undesirable |  |
| 3.24 | Wheelchair | ( ) Essential  ( ) Unessential  ( ) Desirable  ( ) Undesirable | ( ) Essential  ( ) Unessential  ( ) Desirable  ( ) Undesirable | ( ) Essential  ( ) Unessential  ( ) Desirable  ( ) Undesirable | Patient transport |

Other Suggestions:

**PART 4 – MATERIALS**

| **No. of Item** | **Description** | **The evaluation of the expert as to the need for the item with the type of unit** | | | **Role / Use** |
| --- | --- | --- | --- | --- | --- |
|  |  | **Type 1** | **Type 2** | **Type 3** |  |
| 4.01 | Syringes 1 mL | ( ) Essential  ( ) Unessential  ( ) Desirable  ( ) Undesirable | ( ) Essential  ( ) Unessential  ( ) Desirable  ( ) Undesirable | ( ) Essential  ( ) Unessential  ( ) Desirable  ( ) Undesirable | Administration of adrenaline and antihistamines |
| 4.02 | Syringes 3 mL | ( ) Essential  ( ) Unessential  ( ) Desirable  ( ) Undesirable | ( ) Essential  ( ) Unessential  ( ) Desirable  ( ) Undesirable | ( ) Essential  ( ) Unessential  ( ) Desirable  ( ) Undesirable |  |
| 4.03 | Syringes 5-20 mL | ( ) Essential  ( ) Unessential  ( ) Desirable  ( ) Undesirable | ( ) Essential  ( ) Unessential  ( ) Desirable  ( ) Undesirable | ( ) Essential  ( ) Unessential  ( ) Desirable  ( ) Undesirable | Intravenous medicine administration, wound care and other |
| 4.04 | Flexible peripheral venous catheter for children | ( ) Essential  ( ) Unessential  ( ) Desirable  ( ) Undesirable | ( ) Essential  ( ) Unessential  ( ) Desirable  ( ) Undesirable | ( ) Essential  ( ) Unessential  ( ) Desirable  ( ) Undesirable |  |
| 4.05 | Flexible peripheral venous catheter for adults | ( ) Essential  ( ) Unessential  ( ) Desirable  ( ) Undesirable | ( ) Essential  ( ) Unessential  ( ) Desirable  ( ) Undesirable | ( ) Essential  ( ) Unessential  ( ) Desirable  ( ) Undesirable |  |
| 4.06 | Rigid peripheral venous catheter (scalp) for children | ( ) Essential  ( ) Unessential  ( ) Desirable  ( ) Undesirable | ( ) Essential  ( ) Unessential  ( ) Desirable  ( ) Undesirable | ( ) Essential  ( ) Unessential  ( ) Desirable  ( ) Undesirable |  |
| 4.07 | Rigid peripheral venous catheter (scalp) for adults | ( ) Essential  ( ) Unessential  ( ) Desirable  ( ) Undesirable | ( ) Essential  ( ) Unessential  ( ) Desirable  ( ) Undesirable | ( ) Essential  ( ) Unessential  ( ) Desirable  ( ) Undesirable |  |
| 4.08 | Cotton wool | ( ) Essential  ( ) Unessential  ( ) Desirable  ( ) Undesirable | ( ) Essential  ( ) Unessential  ( ) Desirable  ( ) Undesirable | ( ) Essential  ( ) Unessential  ( ) Desirable  ( ) Undesirable | Wound treatment |
| 4.09 | Gauze | ( ) Essential  ( ) Unessential  ( ) Desirable  ( ) Undesirable | ( ) Essential  ( ) Unessential  ( ) Desirable  ( ) Undesirable | ( ) Essential  ( ) Unessential  ( ) Desirable  ( ) Undesirable |  |
| 4.10 | Multi-way or 3-way tap | ( ) Essential  ( ) Unessential  ( ) Desirable  ( ) Undesirable | ( ) Essential  ( ) Unessential  ( ) Desirable  ( ) Undesirable | ( ) Essential  ( ) Unessential  ( ) Desirable  ( ) Undesirable | Intravenous medicine administrations |
| 4.11 | O_2_ catheter | ( ) Essential  ( ) Unessential  ( ) Desirable  ( ) Undesirable | ( ) Essential  ( ) Unessential  ( ) Desirable  ( ) Undesirable | ( ) Essential  ( ) Unessential  ( ) Desirable  ( ) Undesirable | Emergency care |
| 4.12 | Needles (13x4,5) | ( ) Essential  ( ) Unessential  ( ) Desirable  ( ) Undesirable | ( ) Essential  ( ) Unessential  ( ) Desirable  ( ) Undesirable | ( ) Essential  ( ) Unessential  ( ) Desirable  ( ) Undesirable | Preparation of medicines and blood collection |
| 4.13 | Needles (25x7) | ( ) Essential  ( ) Unessential  ( ) Desirable  ( ) Undesirable | ( ) Essential  ( ) Unessential  ( ) Desirable  ( ) Undesirable | ( ) Essential  ( ) Unessential  ( ) Desirable  ( ) Undesirable |  |
| 4.14 | Needles (25x8) | ( ) Essential  ( ) Unessential  ( ) Desirable  ( ) Undesirable | ( ) Essential  ( ) Unessential  ( ) Desirable  ( ) Undesirable | ( ) Essential  ( ) Unessential  ( ) Desirable  ( ) Undesirable |  |
| 4.15 | Medical tape | ( ) Essential  ( ) Unessential  ( ) Desirable  ( ) Undesirable | ( ) Essential  ( ) Unessential  ( ) Desirable  ( ) Undesirable | ( ) Essential  ( ) Unessential  ( ) Desirable  ( ) Undesirable |  |
| 4.16 | Tourniquet for blood collection | ( ) Essential  ( ) Unessential  ( ) Desirable  ( ) Undesirable | ( ) Essential  ( ) Unessential  ( ) Desirable  ( ) Undesirable | ( ) Essential  ( ) Unessential  ( ) Desirable  ( ) Undesirable |  |
| 4.17 | Non-sterile gloves | ( ) Essential  ( ) Unessential  ( ) Desirable  ( ) Undesirable | ( ) Essential  ( ) Unessential  ( ) Desirable  ( ) Undesirable | ( ) Essential  ( ) Unessential  ( ) Desirable  ( ) Undesirable | Personal protective equipment |
| 4.18 | Sterile gloves | ( ) Essential  ( ) Unessential  ( ) Desirable  ( ) Undesirable | ( ) Essential  ( ) Unessential  ( ) Desirable  ( ) Undesirable | ( ) Essential  ( ) Unessential  ( ) Desirable  ( ) Undesirable |  |
| 4.19 | Measuring tape | ( ) Essential  ( ) Unessential  ( ) Desirable  ( ) Undesirable | ( ) Essential  ( ) Unessential  ( ) Desirable  ( ) Undesirable | ( ) Essential  ( ) Unessential  ( ) Desirable  ( ) Undesirable | Clinical evaluation of the lesion |
| 4.20 | Skin marker | ( ) Essential  ( ) Unessential  ( ) Desirable  ( ) Undesirable | ( ) Essential  ( ) Unessential  ( ) Desirable  ( ) Undesirable | ( ) Essential  ( ) Unessential  ( ) Desirable  ( ) Undesirable |  |
| 4.21 | Bandage | ( ) Essential  ( ) Unessential  ( ) Desirable  ( ) Undesirable | ( ) Essential  ( ) Unessential  ( ) Desirable  ( ) Undesirable | ( ) Essential  ( ) Unessential  ( ) Desirable  ( ) Undesirable | Wound treatment |
| 4.22 | Macrodrip IV infusion set | ( ) Essential  ( ) Unessential  ( ) Desirable  ( ) Undesirable | ( ) Essential  ( ) Unessential  ( ) Desirable  ( ) Undesirable | ( ) Essential  ( ) Unessential  ( ) Desirable  ( ) Undesirable | Medicine Administration |
| 4.23 | Microdrip IV infusion set | ( ) Essential  ( ) Unessential  ( ) Desirable  ( ) Undesirable | ( ) Essential  ( ) Unessential  ( ) Desirable  ( ) Undesirable | ( ) Essential  ( ) Unessential  ( ) Desirable  ( ) Undesirable |  |
| 4.24 | Scalpel blade | ( ) Essential  ( ) Unessential  ( ) Desirable  ( ) Undesirable | ( ) Essential  ( ) Unessential  ( ) Desirable  ( ) Undesirable | ( ) Essential  ( ) Unessential  ( ) Desirable  ( ) Undesirable | Clinical procedures |
| 4.25 | Oxygen mask (continuous nebulization) | ( ) Essential  ( ) Unessential  ( ) Desirable  ( ) Undesirable | ( ) Essential  ( ) Unessential  ( ) Desirable  ( ) Undesirable | ( ) Essential  ( ) Unessential  ( ) Desirable  ( ) Undesirable | Emergency care |
| 4.26 | Disposable surgical mask | ( ) Essential  ( ) Unessential  ( ) Desirable  ( ) Undesirable | ( ) Essential  ( ) Unessential  ( ) Desirable  ( ) Undesirable | ( ) Essential  ( ) Unessential  ( ) Desirable  ( ) Undesirable | Personal protective equipment |
| 4.27 | Suture Kit (scissors, tweezers, nylon thread 3-0 / cotton 0,2, scalpel) | ( ) Essential  ( ) Unessential  ( ) Desirable  ( ) Undesirable | ( ) Essential  ( ) Unessential  ( ) Desirable  ( ) Undesirable | ( ) Essential  ( ) Unessential  ( ) Desirable  ( ) Undesirable | Wound treatment |
| 4.28 | Penrose drain | ( ) Essential  ( ) Unessential  ( ) Desirable  ( ) Undesirable | ( ) Essential  ( ) Unessential  ( ) Desirable  ( ) Undesirable | ( ) Essential  ( ) Unessential  ( ) Desirable  ( ) Undesirable |  |

Other Suggestions:

**PART 5 – MEDICINE**

| **No. of Item** | **Description** | **The evaluation of the expert as to the need for the item with the type of unit** | | | **Role / Use** |
| --- | --- | --- | --- | --- | --- |
|  |  | **Type 1** | **Type 2** | **Type 3** |  |
| 5.01 | Corticosteroids | ( ) Essential  ( ) Unessential  ( ) Desirable  ( ) Undesirable | ( ) Essential  ( ) Unessential  ( ) Desirable  ( ) Undesirable | ( ) Essential  ( ) Unessential  ( ) Desirable  ( ) Undesirable | Premedication and treatment of adverse reactions |
| 5.02 | Anti-histamines | ( ) Essential  ( ) Unessential  ( ) Desirable  ( ) Undesirable | ( ) Essential  ( ) Unessential  ( ) Desirable  ( ) Undesirable | ( ) Essential  ( ) Unessential  ( ) Desirable  ( ) Undesirable |  |
| 5.03 | Adrenaline | ( ) Essential  ( ) Unessential  ( ) Desirable  ( ) Undesirable | ( ) Essential  ( ) Unessential  ( ) Desirable  ( ) Undesirable | ( ) Essential  ( ) Unessential  ( ) Desirable  ( ) Undesirable | Treatment of adverse reactions |
| 5.04 | Painkillers | ( ) Essential  ( ) Unessential  ( ) Desirable  ( ) Undesirable | ( ) Essential  ( ) Unessential  ( ) Desirable  ( ) Undesirable | ( ) Essential  ( ) Unessential  ( ) Desirable  ( ) Undesirable | Pain management |
| 5.05 | Opioids | ( ) Essential  ( ) Unessential  ( ) Desirable  ( ) Undesirable | ( ) Essential  ( ) Unessential  ( ) Desirable  ( ) Undesirable | ( ) Essential  ( ) Unessential  ( ) Desirable  ( ) Undesirable |  |
| 5.06 | Diuretic | ( ) Essential  ( ) Unessential  ( ) Desirable  ( ) Undesirable | ( ) Essential  ( ) Unessential  ( ) Desirable  ( ) Undesirable | ( ) Essential  ( ) Unessential  ( ) Desirable  ( ) Undesirable | Stimulation of diuresis |
| 5.07 | Saline 0.9% | ( ) Essential  ( ) Unessential  ( ) Desirable  ( ) Undesirable | ( ) Essential  ( ) Unessential  ( ) Desirable  ( ) Undesirable | ( ) Essential  ( ) Unessential  ( ) Desirable  ( ) Undesirable | Hydration |
| 5.08 | Glucose solution 5% | ( ) Essential  ( ) Unessential  ( ) Desirable  ( ) Undesirable | ( ) Essential  ( ) Unessential  ( ) Desirable  ( ) Undesirable | ( ) Essential  ( ) Unessential  ( ) Desirable  ( ) Undesirable |  |
| 5.09 | Antibiotic | ( ) Essential  ( ) Unessential  ( ) Desirable  ( ) Undesirable | ( ) Essential  ( ) Unessential  ( ) Desirable  ( ) Undesirable | ( ) Essential  ( ) Unessential  ( ) Desirable  ( ) Undesirable | Treatment of secondary infection |
| 5.10 | Sedatives | ( ) Essential  ( ) Unessential  ( ) Desirable  ( ) Undesirable | ( ) Essential  ( ) Unessential  ( ) Desirable  ( ) Undesirable | ( ) Essential  ( ) Unessential  ( ) Desirable  ( ) Undesirable | Auxiliary treatment |
| 5.11 | Topical anesthetics | ( ) Essential  ( ) Unessential  ( ) Desirable  ( ) Undesirable | ( ) Essential  ( ) Unessential  ( ) Desirable  ( ) Undesirable | ( ) Essential  ( ) Unessential  ( ) Desirable  ( ) Undesirable |  |
| 5.12 | Antiseptics | ( ) Essential  ( ) Unessential  ( ) Desirable  ( ) Undesirable | ( ) Essential  ( ) Unessential  ( ) Desirable  ( ) Undesirable | ( ) Essential  ( ) Unessential  ( ) Desirable  ( ) Undesirable | Wound treatment |

Other Suggestions:
