# Supplementary material for: Development and validation of a minimum requirements checklist for snakebite envenoming treatment in the Brazilian Amazonia
Source: PLoS Negl Trop Dis. 2024 Jan 19;18(1):e0011921. doi: 10.1371/journal.pntd.0011921 (PMC10829989; doi:10.1371/journal.pntd.0011921)
Supplement: Attachment — Submitted filename: PONE-D-23-32936_reviewer.pdf [file pntd.0011921.s006.pdf]

PLOS ONE

Knowledge, Attitude, and Practices of Snakes, Snakebites and their Management among Health Workers in Sudan: Need for Internet-mediated Knowledge Management System for Continuing Professional Development.

--Manuscript Draft--

Manuscript Number:	PONE-D-23-32936
Article Type:	Research Article
Full Title:	Knowledge, Attitude, and Practices of Snakes, Snakebites and their Management among Health Workers in Sudan: Need for Internet-mediated Knowledge Management System for Continuing Professional Development.
Short Title:	Snakes, Snakebites and their Management in Sudan: Need for Continuing Professional Development
Corresponding Author:	Ali Awadallah Saeed<br>National University - Sudan<br>Khartoum, SUDAN
Keywords:	Snakes<br>Snakebites<br>Health Workers<br>Internet-mediated Knowledge<br>Continuing Professional Development.
Abstract:	Background<br><br>Snakebite is considered one of the world’s most serious medical problems, and the WHO recently declared snakebite envenomation a neglected tropical disease and called for new strategies to combat it and resources to implement them.<br><br>Material and methods<br><br>This descriptive cross-sectional survey was conducted in August 2022. It included 394 medical and health care providers from different parts of Sudan. The questionnaire contains seven different sections included important and basic knowledge of snakes, snake bites and their management. The data were entered into Microsoft Excel and managed by the Statistical Package for Social Sciences (SPSS) version 20 (IBM SPSSInc., Chicago, IL).<br><br>Results<br><br>394 medical and health care providers participated, of them; there were 44.7% males, and 53.3% females. Only 25.9% participants had training in snake bite management, 58.1% answered correctly snake knowledge questions 45.3% answered the snakebite knowledge questions correctly, and 60.4% answered snakebite practice questions correctly. Only 14% had rated themselves as very confident in the management of snakebites, 40.9% stated their health facility has a protocol for managing snakebites.<br><br>Conclusion<br><br>Intensive educational effort should be focused on basic knowledge of snakes, snakebites, prevention, management of snakebites and first aid measures. With the advent of the internet and tools such as learning management systems, introducing and disseminating knowledge has become easier and faster. Hence, we proposed an internet-mediated Knowledge Management System for Continuing Professional Development.
Order of Authors:	Ali Awadallah Saeed
	Ahmed Hassan Fahal
	Omer Abdelhamid Gibreel

Powered by Editorial Manager® and ProduXion Manager® from Aries Systems Corporation

	Ayman B. Mousa
	Saeed M Omer
	Abdallateif Alkhair Omer
	Intisar A. M. A. Elalawy
**Additional Information:**	
**Question**	**Response**
**Financial Disclosure**<br><br>Enter a financial disclosure statement that describes the sources of funding for the work included in this submission. Review the submission guidelines for detailed requirements. View published research articles from *PLOS ONE* for specific examples.<br><br>This statement is required for submission and **will appear in the published article** if the submission is accepted. Please make sure it is accurate.<br><br>**Funded studies**<br>Enter a statement with the following details:<br>• Initials of the authors who received each award<br>• Grant numbers awarded to each author<br>• The full name of each funder<br>• URL of each funder website<br>• Did the sponsors or funders play any role in the study design, data collection and analysis, decision to publish, or preparation of the manuscript?<br><br>Did you receive funding for this work?	The author(s) received no specific funding for this work.
**Competing Interests**<br><br>Use the instructions below to enter a competing interest statement for this submission. On behalf of all authors, disclose any competing interests that could be perceived to bias this work—acknowledging all financial support and any other relevant financial or non-financial competing interests.	NO authors have competing interests

This statement is required for submission and **will appear in the published article** if the submission is accepted. Please make sure it is accurate and that any funding sources listed in your Funding Information later in the submission form are also declared in your Financial Disclosure statement.<br><br>View published research articles from *PLOS ONE* for specific examples.<br><br>**NO authors have competing interests**<br><br>Enter: *The authors have declared that no competing interests exist.*<br><br>**Authors with competing interests**<br><br>Enter competing interest details beginning with this statement:<br><br>*I have read the journal's policy and the authors of this manuscript have the following competing interests: [insert competing interests here]*<br><br>* typeset	
**Ethics Statement**<br><br>Enter an ethics statement for this submission. This statement is required if the study involved:<br><br>• Human participants<br>• Human specimens or tissue<br>• Vertebrate animals or cephalopods<br>• Vertebrate embryos or tissues<br>• Field research<br><br>Write "N/A" if the submission does not require an ethics statement.<br><br>General guidance is provided below. Consult the submission guidelines for detailed instructions. **Make sure that all information entered here is included in the Methods section of the manuscript.**	National Uinversity Research Ethics Committee (NU-REC), Approval Number NU-REC/11-018/12

**Format for specific study types**<br><br>**Human Subject Research (involving human participants and/or tissue)**<br>• Give the name of the institutional review board or ethics committee that approved the study<br>• Include the approval number and/or a statement indicating approval of this research<br>• Indicate the form of consent obtained (written/oral) or the reason that consent was not obtained (e.g. the data were analyzed anonymously)<br><br>**Animal Research (involving vertebrate animals, embryos or tissues)**<br>• Provide the name of the Institutional Animal Care and Use Committee (IACUC) or other relevant ethics board that reviewed the study protocol, and indicate whether they approved this research or granted a formal waiver of ethical approval<br>• Include an approval number if one was obtained<br>• If the study involved *non-human primates*, add *additional details* about animal welfare and steps taken to ameliorate suffering<br>• If anesthesia, euthanasia, or any kind of animal sacrifice is part of the study, include briefly which substances and/or methods were applied<br><br>**Field Research**<br><br>Include the following details if this study involves the collection of plant, animal, or other materials from a natural setting:<br>• Field permit number<br>• Name of the institution or relevant body that granted permission	
**Data Availability**<br><br>Authors are required to make all data underlying the findings described fully available, without restriction, and from the time of publication. PLOS allows rare exceptions to address legal and ethical concerns. See the PLOS Data Policy and FAQ for detailed information.	Yes - all data are fully available without restriction

A Data Availability Statement describing where the data can be found is required at submission. Your answers to this question constitute the Data Availability Statement and **will be published in the article**, if accepted.<br><br>**Important:** Stating 'data available on request from the author' is not sufficient. If your data are only available upon request, select 'No' for the first question and explain your exceptional situation in the text box.<br><br>Do the authors confirm that all data underlying the findings described in their manuscript are fully available without restriction?	
**Describe where the data may be found in full sentences. If you are copying our sample text, replace any instances of XXX with the appropriate details.**<br><br>• If the data are **held or will be held in a public repository**, include URLs, accession numbers or DOIs. If this information will only be available after acceptance, indicate this by ticking the box below. For example: *All XXX files are available from the XXX database (accession number(s) XXX, XXX.).*<br>• If the data are all contained **within the manuscript and/or Supporting Information files**, enter the following: *All relevant data are within the manuscript and its Supporting Information files.*<br>• If neither of these applies but you are able to provide **details of access elsewhere**, with or without limitations, please do so. For example:<br><br>*Data cannot be shared publicly because of [XXX]. Data are available from the XXX Institutional Data Access / Ethics Committee (contact via XXX) for researchers who meet the criteria for access to confidential data.*<br><br>*The data underlying the results presented in the study are available*	All relevant data are within the manuscript and its Supporting Information files.

from (include the name of the third party and contact information or URL).<br>• This text is appropriate if the data are owned by a third party and authors do not have permission to share the data.<br><br>* typeset	
Additional data availability information:	

Powered by Editorial Manager® and ProduXion Manager® from Aries Systems Corporation

Manuscript

Click here to access/download;Manuscript;R1 Manuscript KAP snakes (1).docx

Knowledge, Attitude, and Practices of Snakes, Snakebites and their Management among Health Workers in Sudan: Need for Internet-mediated Knowledge Management System for Continuing Professional Development.

Ali Awadallah Saeed[1, 2], Ahmed Hassan Fahal[2], Omer Abdelhamid Gibreel[3], Ayman B. Mousa[1], Saeed M Omer [4], Abdallateif Alkhair Omer [5], Intisar A. M. A. Elalawy [6]

1 Department of Pharmacology and therapeutics, Faculty of Clinical and Industrial Pharmacy, National University-Sudan, E-mail: aliawadsaeed@nu.edu.sd , ORCID: https://orcid.org/0000-0003-3524-4825, Scopus ID: https://www.scopus.com/authid/detail.uri?authorId=58106128900

2 Mycetoma Research Center, University of Khartoum

3 Department of Management Information System, College of Business Administration, Gulf University for Science and Technology – Gibreel.o@gust.edu.kw

4 Faculty of Medicine and Health Science, Gadarif University, Sudan, drsaeedomar@yahoo.com

5 Faculty of Medicine, Omdurman Islamic University, abdallateif.1983@gmail.com

6 Alrayan College of Health Sciences and Nursing, Pharmacy program, Almadina, Saudi Arabia, ORCID: 0009-0002-1777-1058, E-mail: intisarelalawy@gmail.com

Corresponding author: Ali Awadallah Saeed

aliawadsaeed@nu.edu.sd

Abstract

Background:

Snakebite is considered one of the world's most serious medical problems, and the WHO recently declared snakebite envenomation a neglected tropical disease and called for new strategies to combat it and resources to implement them.

Material and methods

This descriptive cross-sectional survey was conducted in August 2022. It included 394 medical and health care providers from different parts of Sudan. The questionnaire contains seven different sections included important and basic knowledge of snakes, snake bites and their management. The data were entered into Microsoft Excel and managed by the Statistical Package for Social Sciences (SPSS) version 20 (IBM SPSSInc., Chicago, IL).

Results

394 medical and health care providers participated, of them; there were 44.7% males, and 53.3% females. Only 25.9% participants had training in snake bite management, 58.1% answered correctly snake knowledge questions 45.3% answered the snakebite knowledge questions correctly, and 60.4% answered snakebite practice questions correctly. Only 14% had rated themselves as very confident in the management of snakebites, 40.9% stated their health facility has a protocol for managing snakebites.

Conclusion:

Intensive educational effort should be focused on basic knowledge of snakes, snakebites, prevention, management of snakebites and first aid measures. With the advent of the internet and tools such as learning management systems, introducing and disseminating knowledge has become easier and faster. Hence, we proposed an internet-mediated Knowledge Management System for Continuing Professional Development.

Introduction

Snakebite is considered one of the world's most serious medical problems, and the WHO recently declared snakebite envenomation a neglected tropical disease (9) and called for new strategies to combat it and resources to implement them (10). There are 5.4 million poisonous snake bites globally, fatal in around 2.5 million bites. Snake bites are accountable for 1,25,000 deaths annually. [(1)] WHO reported that 81-95% of snake bites occur in the tropical regions of South Asia, South-East Asia, Sub-Saharan Africa and Latin America. [(2)] In sub-Saharan Africa, the number of persons treated in health centers for snakebite envenomation is estimated at 315,000 cases per year, with more than 9,000 amputations and 7,000 deaths. Since many victims do not seek medical care and they are invisible to official reports thus, the actual statistics of snake envenomation remain underestimated. (8).

In some parts of the African continent, the snakebites' burden is higher than that of trypanosomiasis, leishmaniasis, and onchocerciasis, and its mortality can be higher than malaria. The snakebites' high mortality in Africa is multifactorial, including lack of antitoxins, inadequate health care system, and scarcity of rapid access to medical centers.[3] Furthermore, many

inhabitants of rural areas believe in traditional healers and do not seek treatment due to low socioeconomic and health education levels and victims died shortly after the snakebite accident, and that is not reported in the hospital records. In rural regions, where most snakebite occur (8), the general population's awareness of appropriate behavior in response to snakebites is poor. A study in Nigeria showed that most patients had attempted at least one potentially deleterious first aid measure prior to presentation to hospitals. [(11)] In Ghana, an intervention to improve snakebite medical care ability and adherence to protocols led to a significant improvement in care and decreased significantly the snakebite mortality post the intervention. [(12)]

In Sudan, snakebite statistics are lacking, despite the high estimated burden of the problem. [6] During the last 70 years, only a few data were reported on the Sudanese venomous snakes. One study reported the presence of 17 medically significant snakes belonging to three major families: Burrowing asps, Elapidae, and Viperidae. These snakes usually become abundant during and after the rainy season, and most snakebite victims are farm workers.[5] The records of the Natural History Museum, University of Khartoum, since the early last century, revealed some interesting data on snakes in Sudan. [5,7]

To determine the poor outcome of snakebite management in Sudan, the present study was set out to determine the medical and health care providers' knowledge, attitude and practice of snakebites management. Furthermore, the study proposed that need to introduce an internet based learning management system (LMS) to support the dissemination of knowledge and practices on snakes bite management. There is proposal to use of internet based learning management system to increase awareness about snakebites due to the success of this form of internet-enabled learning in other part of Africa (23-28). For example, South Africa used internet-mediated learning management system to tackle the issue of AIDS (24), In Libera an internet-based learning management system was introduced after the Ebola virus epidemic (23).

Material and methods

This descriptive cross-sectional survey was conducted in August 2022. It included 394 medical and health care providers from different parts of Sudan. A validated web-based self-administrated questionnaire was used to collect the required data. The questionnaire included important and basic knowledge of snakes, snake bites and their management. A pilot study was conducted prior to the study to validate the questionnaire. The questionnaire was distributed widely online to medical and health care providers. They gave informed consent.

Sudan Snake Bite Research Network prepared this questionnaire. It contains seven different sections: section one comprised of seven socio-demographic questions on the study participants, section two had eight questions on the participants' knowledge of snakes, section three contained nine questions on knowledge of snakebites, section four contained five questions regarding practice towards snakebites, section five contained two questions regarding attitude toward snakebites, section six had seven questions regarding snakebites management and section seven included ten questions on snakebites management knowledge.

Data analysis

The data were entered into Microsoft Excel and managed by the Statistical Package for Social Sciences (SPSS) version 20 (IBM SPSSInc., Chicago, IL). Frequency and percentage were calculated for qualitative variables, and median and interquartile range (IQR) were calculated for quantitative variables. A total score of knowledge, attitude, and practice was calculated and later classified into good and poor based on the average score. Attitudes to dealing with snake bites were expressed in%. Chi-square was performed to find relationships between good knowledge, practice, and other selected variables. Questions were analyzed to determine the knowledge and practice levels. Each correct answer was given a score of 1, and the "don't know", and "wrong" answers were given 0 points. The result is expressed as%. The results were rated good and bad based on the median.

Ethical considerations

Informed oral consent from all study subjects was obtained, and confidentiality was maintained

Results

The study included 394 medical and health care providers, of them, there were 176 (44.7%) males, and 218 (53.3%) females, 142 (36%) were less than 40 years old, and 252(64%) aged were more than that. The study participants were pharmacists 122 (31%), medical officers 94 (23.9%), registrars 79 (20%), house officers 46 (11.7%) nurse 27 (6.8%) and consultant 26(6.6%). The study showed that 255 participants (64.7%) had less than five years of medical and health experience, 89 (22.3%) had experience ranging between five and ten years, and 50 (13%) had more than ten years’ experience. Only 102 participants (25.9%) had training in snake bite management, gained from senior colleagues (37%), 28.5% were self-educated, and 10% gained it during training. (Table 1)

There were eight questions on snakes knowledge, and the results showed that 321 (81.5) corrected answered the fact that not all snakes are poisonous, 129 (32.7) stated all snakes are carnivorous, 255 (64.7) said not all snakes are venomous, 113 (28.7) recognized all snakes has fangs in front of their mouth, 213 (54.1) thought snakes pick sounds using their ears, 213 (54.1%) believe snakes are important for farmers and 255 (64.7%) believed deforestation and urbanization have increased human-snake interaction. (Table 2) The study showed that 322 participants (81.7) knew snakes are reptiles. (Table 3) The study showed that 229 participants (58.1%) answered correctly snake knowledge questions while 165 (41.9%) had incorrect answers.

Regarding snakebites' knowledge, there were nine questions. 85 (21.6%) of the participants responded correctly to the question on handling a dead snake's head is not safe enough, 195 (49.5%) recognized the fact that marks can always be seen or found on the victim after every snake bite, 219 (55.6%) believed that a person reported at the hospital with symptoms of snakebite can be given antitoxin injection without actually being bitten by a snake, 122 (31%) believed sleeping under mosquito nets can prevent snakebites. A venomous ("poisonous") snakes always inject venom (poison) into the victim; 151 (38.3%) do not believe this statement, 316 (80.2%) recognized that the snake type determines the symptoms and signs of snakebites and 279 (70.8%) thought that the symptoms and signs of the snakebite depend on the amount of venom injected by the snake. (Table 2)

209 (53%) believed the symptoms and signs could determine the snake and venom injected, and 31 (7.9%) felt the snakebite common occurs during the day. (Table 3) For the snakebites knowledge, 179 participants (45.3%) answered the snakebite knowledge questions correctly, while 215 (54.7%) responded incorrectly.

The obtained data showed that 247 participants (26.7%) believed that a tourniquet prevents the spread of poison in the remaining part of the body, and 320 (81.2%) stated reassurance of the victim should be practice as 1st protocol for prevention. Regarding the incision at the bite site helps remove that poison, that was the belief of 186 participants (47.2%), and 282 (71.6%) thought if the leg is the bite site, its elevation will not reduce poison spread. Bringing the snake to the treating physician increases the chances of survival by correct identification of the snake believed by 297 participants (75.4%). (Table 4)

In this study, 238 participants (60.4%) answered snakebite practice questions correctly, while 156 (39.6%) did not.

Regarding snakebites' attitude, residing in cities is a protective factor against snakebites was stated by 297 participants (75.4%), and 106 (26.9%) said that the snakebite is the outcome of revenge inspired by past incidents. (Table 5)

The study showed only 55 participants (14%) had rated themselves as very confident in the management of snakebites, and 203 participants (51.5%) had previously managed, supervised or nursed a snakebite patient. Regarding management and help provided by participants in snakebite management in 2021, 20 (5.1%) managed more than 100 patients, and 56 (14.2%) managed between 10 to 50 patients. 161 (40.9%) stated their health facility has a protocol for managing snakebites. Only 179 participants (45.4%) said their health facility has what it takes to effectively manage snake bites. (Table 6)

Regarding the management of snakebites, 265 participants (67.3) considered it an emergency. Most of the participants, 178 (45.2%) manage the snakebite by admission and treatment rather than referring patients immediately 26 (6.6%). Sixty-one patients (15.5%) correctly said a 20-minute whole blood count test is the most appropriate test for snakebites management. Antivenoms are the only antidotes in managing snakebites by venomous snakes, as stated by 267 participants (75.4%).

Antivenoms made anywhere in the world are good for the management of snake bite victims, was said by 199 participants (50.5%), and 191 (48.4) believed that antivenoms should be given to all patients bitten by snakes. Fifty-seven participants (14.5%) thought that the intramuscular injection of an anti-snake venom is effective as an intravenous one. One hundred ninety-seven participants (50%) did not know how long the anti-snake venoms could remain useful after the expiry date. In using anti-snake venoms, it is better to give low doses repeated over several days than high initial doses 132 participants (33.6%) believed that is the correct regimen.

Discussion:

In a study conducted by Subedi et al among medical students 50.7% were males and 49.3% females (13), in study conducted by Amritesh Kumar was similar 14, 52% males and 48% females, in study conducted by Kashif Ali and et al 58% were males and 42% were females (21), were as in our study were 44.7% males, and 53.3% females.

In this study 37% participants get their knowledge and skill in snake bite management from their senior colleges, 28.5% self-educated and 24.3% don't know the skills.

In Kumar and et al study (14), the knowledge domain less than half i.e. (48%) of respondents had good knowledge, which is nearby to our findings 58.1% answered correctly.

100% of the study participants knew that all snakes are not poisonous as stated by Kashif Ali and et al (21). In study conducted by Kumar A and et al, majority of respondents i.e. 88 % correctly responded that all snakes are not poisonous (14), which similar to our study 81.5% respondent not all snakes are not poisonous.

In the Kumar A and et al study (14), less than half (40%) had knowledge that snake bites were fatal, which is less than our findings, more than half (64.7%) said not all snakes are venomous.

More than half i.e. (55%) agreed about the fact that snakes were important for farmers in Kumar A and et al study (14), and this is similar to our findings, 54.1% believe snakes are important for farmers, while 94% of the study participants by Kashif Ali and et al believed that snakes are helpful to farmers (21).

In Kumar a and et al study (14), majority (82%) responded correctly that deforestation and urbanization had increased human-snake interaction, which is high compared to our findings, 64.7% believed deforestation and urbanization have increased human-snake interaction.

In Kumar and et al study (14), tow third (66%) stated correctly that snakes has fangs in front of their mouth, which high compared to our findings 28.7%.

In area regarding snakebites knowledge, 45.3% participants answered the snakebite knowledge questions correctly, while 54.7% responded incorrectly.

Only 21.6% participants stated handling a dead snake's head is not safe enough, which consistent to WHO guidelines, do not handle the snake with your bare hands as even a severed head can bite. (17)

A venomous ("poisonous") snakes always inject venom (poison) into the victim; 38.3% do not believe this statement, 80.2% recognized that the snake type determines the symptoms and signs of snakebites and 70.8% thought that the symptoms and signs of the snakebite depend on the amount of venom injected by the snake. 53% believed the symptoms and signs could determine the snake and venom injected.

Only 7.9% felt the snakebite common occurs during the day, compared to (92%) participants believed that snakes bite mostly during monsoons and at night (21).

In Kumar and et al study (14), in snakebites practice domain and after answering practice questions, only 35 % of respondent had good practice, this low compared to that found in our study, more than half (60.4%) answered snakebite practice questions correctly.

Only 26.7% in this study believed that a tight band (tourniquet) prevents the spread of poison in the remaining part of the body, when compared to study conducted in Srilanka by Anjana et al (15), application of tight band (tourniquet) proximal to the site of bite was considered as an important first aid measure among 74.9% of cases where as it was higher (96%) in a study conducted by Kumar et al. (16) and (89%) in Kumar A et al study (14), compared to study by Kashif Ali, all the study participants said they would tie a tourniquet at the site and would rush to the nearest health facility (21)

In practice domain 89% of inters responded correctly in Kumar and et study (14) that more than half of interns i.e. (59%) responded that practice of reassurance as the first protocol in snake bite management, which is low compared to our findings 81.2% stated reassurance of the victim should be practice as first protocol for prevention.

Bringing snakes to physician in Kumar and et al study (14) , helped in correct identification of species and better patient management were recommended by more than half i.e. 54% which is low compared to our study, three quarter (75.4%). Killed snake should be taken to the dispensary or hospital with the patient in case it can be identified as stated by WHO (17).

The incision at the bite site helps remove that poison correctly answered by 47.2%, which is high and douple to that found by Kumar and et al study (14), 24%. In our findings, 28.4% from respondent correctly answered elevation of leg will reduce poison spread, which is low compared to 62% found by Kumar and et al study. (14)

In attitude domain regarding snakebites attitude, Kumar and et al (14) found almost more than 8% of the interns stated right attitude, that snake bites were outcome of revenge inspired from past incidents and residing in cities is a protective factor from snake bite, which is very low compared to our findings, residing in cities is a protective factor against snakebites was stated by 75.4%, and 26.9% said that the snakebite is the outcome of revenge inspired by past incidents.

In management of snakebites, the study showed only 14% participants had rated themselves as very confident in the management of snakebites, and 51.5% participants had previously managed, supervised or nursed a snakebite patient. Regarding management and help provided by

participants in snakebite management in 2021, 5.1% managed more than 100 patients, and 14.2% managed between 10 to 50 patients.

40.9% stated their health facility has a protocol for managing snakebites. Only 45.4% participants said their health facility has what it takes to effectively manage snake bites.

Regarding the management of snakebites, 67.3% participants considered it an emergency. Most of the participants, 178 (45.2%) manage the snakebite by admission and treatment rather than referring patients immediately (6.6%). The patient must be transported to a place where they can receive medical care (dispensary or hospital) as quickly, but as safely and comfortably, as possible as stated by WHO (17).

As stated by WHO guidelines for the management of snakebites (17), 20-minute whole blood clotting test (20WBCT) is most common and very useful and informative laboratory test, in our study only sixty-one patients (15.5%) correctly said a 20-minute whole blood count test is the most appropriate test for snakebites management.

In our study 75.4% participants antivenom is the only specific antidote to snake venom, this consistent with WHO guidelines (17).

50.5% participants stated antivenoms made anywhere in the world are good for the management of snake bite victims. Medical treatment is available for snakebite as stated by all participants and 86% participant knew about availability of treatment for snakebite at PHC study conducted by Kashif Ali and et al (21)

Nearly half of participants (48.4%) believed that antivenoms should be given to all patients bitten by snakes, while in WHO guidelines, antivenom should be given only to patients in whom its benefits are considered likely to exceed its risks, since antivenom is relatively costly and often in limited supply, it should not be used indiscriminately. The risk of reactions should always be taken into consideration [level of evidence E]. (17)

Only 14.5% thought that the intramuscular injection of an anti-snake venom is effective as an intravenous one which is consistent to WHO guidelines, intramuscular blood levels of antivenom never reach those achieved rapidly by intravenous administration (17).

Half of participants (50%) did not know how long the anti-snake venoms could remain useful after the expiry date. Anti-snake venoms can be expected to retain useful activity for months or even years after these dates if stored properly (17). Expired antivenoms could provide an alternative option for snakebite treatment (22).

In using anti-snake venoms, it is better to give low doses repeated over several days than high initial doses, 33.6% believed that is the correct regimen which is not consistent to practice. The recommended dose is often the amount of antivenom required to neutralize the average venom yield when captive snakes are milked of their venom, in practice, the choice of an initial dose of antivenom is usually empirical as stated by WHO (17).

Clinical implication of the study:

In Sudan surveillance of snakebites is essential to understand their epidemiology better and reporting of cases must be improved to provide accurate data and to facilitate stock management, training implementation, and appropriate interventions. Now that snakebites are recognized as a Neglected Tropical Disease, a global, adapted, and sustainable approach is required. Training of health care personnel represents a vital aspect of this global approach.

Technological Implication of the Study:

Due to the introduction of mobile systems in Africa and the recent development of undersea fiber optics cables, the barrier to accessing the internet-mediated knowledge management system has been dramatically reduced. The development of an internet-mediated knowledge management system that provides a repository of lessons learned and best practices with a combination of video lectures and online material (19, 20) is best way to increase knowledge regarding snakes, snakebites and their Management among health workers in Sudan.

Strength and weakness of our study:

The main strength of the current study is the sample size of healthcare workers participated in the study from areas affected by snakebites (394). In addition, we have conducted a multicenter study in six hospitals from both the public and private sectors. While the limitation include, the lack of long-term evaluation of the knowledge, and exact evaluation of healthcare facilities that recipe patients affected by snakebites.

Conclusion

There is a strong need to develop an internet-mediated knowledge management system that would help in Sudan's continuing professional development process in different areas especially in snake, snakebites and management. Recent studies have shown the effectiveness and success of the learning management system (LMS) in Sudan (18).

The present study shown a gap in the knowledge on the snake, snakebite and its management, which should be addressed in different levels, personal, community and at governmental level.

This area should be an important part of medical curriculum for all health professionals because they are exposed to such cases in practice.

Poor training in snake bite management, snake and snakebite knowledge, snakebite practice and lack of health facility protocols for management of snakebites management are major concerns.

Future prospects:

In order to address these issues we suggest that an intensive educational effort should be focused on basic knowledge of snakes, snakebites, prevention, management of snakebites and first aid measures.

Conflict of interest: None

Source of funding: Self-funded

References:

1. Ch(1)ippaux JP. Snake bites: appraisal of the global situation. Bull world health organ. 1998;(5):515-24.
2. Kasturiratne A, Wickremasinghe AR, de Silva N, Gunawardena NK, Pathmeswaran A, Premaratna R, et al. The global burdedn of snake bite: a literature analysis and modeling based on regional estimates of envenoming and deaths. PloS Med. 2008; 5:218.
3. Huda Khalid, Rasha S Azrag, Retrospective hospital-based study on snakebite envenomation in Sudan, Transactions of The Royal Society of Tropical Medicine and Hygiene, Volume 115, Issue 9, September 2021, Pages 992–997, https://doi.org/10.1093/trstmh/trab085
4. Warrell DA. Snake Bite. lancet. 2010; 375:77-88.
5. Kirk R., Poisonous snakes of the Sudan, Sudan Wild life and sport, 1951, vol. II: 17- 27.
6. Kasturiratne A, Wickremasinghe AR, de Silva N et al., The global burden of snakebite: a literature analysis and modeling based on regional estimates of envenoming and deaths, PLoS Med 2008; 5:e218.
7. Corkill NL., A guide for the species represented in the collection of natural history museum Khartoum. Natural History Museum Publication no. 3, 1935.
8. Chippaux JP. Estimate of the burden of snakebites in sub-Saharan Africa: a meta-analytic approach. Toxicon. 2011; 57(4):586–99. https://doi.org/10.1016/j.toxicon.2010.12.022 PMID: 21223975.

9. Organisation WH. Available at http://www.who.int/neglected diseases/diseases/en/. Accessed on December 2017. Neglected tropical diseases.
10. Chippaux JP. Snakebite envenomation turns again into a neglected tropical disease! J Venom Anim Toxins Incl Trop Dis. 2017; 23:38. https://doi.org/10.1186/s40409-017-0127-6 PMID: 28804495; PubMed Central PMCID: PMCPMC5549382.
11. Habib AG. Public health aspects of snakebite care in West Africa: perspectives from Nigeria. J Venom Anim Toxins Incl Trop Dis. 2013; 19(1):27. https://doi.org/10.1186/1678-9199-19-27 PMID: 24134780; PubMed Central PMCID: PMCPMC3831819.
12. Visser LE, Kyei-Faried S, Belcher DW. Protocol and monitoring to improve snake bite outcomes in rural Ghana. Trans R Soc Trop Med Hyg. 2004; 98(5):278–83. https://doi.org/10.1016/S0035-9203(03) 00065-8 PMID: 15109550.
13. Subedi N, Paudel IS, Khadka A, Shrestha U, Mallik VB, Ankur KC. Knowledge of first aid methods and attitude about snake bite among medical students: a cross sectional observational study. J Occup Med Toxicol. 2018;13:26.
14. Kumar A, Gopakumar S, Sathyan S, Singh SB, Bhasy BI. A KAP study on snakebite and its management among medical interns in a tertiary care medical college and hospital in Trivandrum district, Kerala. Int J Community Med Public Health 2019;6:1340-4
15. Silva A, Marikar F, Murugananthan A, Agampodi S. Awareness and perception on prevention, first aid and treatment of snake bite among Srilankan farmers. J Occ Med Toxicol. 2014;9:20.
16. Kumar A, Dasgupta A, Biswas D, Sahoo SKS, Preeti PS. Knowledge regarding snake bite in snake bite in rural Bengal-Are they still lingering on yths and misconceptions? IAIM. 2015;2(7);36-41.
17. David A Warrell, Guidelines for the management of snake-bites, WHO Library Cataloguing-in-Publication data, ISBN 978-92-9022-377-4.
18. Gibreel, O. A., Mahdi, M. A., Saeed, A. A. (2021): E-learning and the Digital Divide Role in Transforming Higher Education in Sudan: An Exploratory Case Study Analysis, Vol 9, No. 1/2, pp. 95 -108.
19. Ahmed, A. and Gibreel, O. (2021), "The role of social media in strengthening civic engagement in the Middle East and North Africa", World Journal of Entrepreneurship,

Management and Sustainable Development, Vol. 17 No. 3, pp. 309-317. https://doi.org/10.1108/WJEMSD-02-2021-0020

20. Gibreel, Omer; Jeon, Seongmin; and Yoo, Byungjoon, "An Intercountry Analysis on the Factors and Barriers to Internet Accessibility", (2013). PACIS 2013 Proceedings. 6. http://aisel.aisnet.org/pacis2013/6
21. Kashif Ali, Ishan Pathak, Knowledge, Attitude and Practice regarding Snakes and Snakebite among Interns, Indian Journal of Forensic and Community Medicine, October-December 2017;4(4):229-231.
22. Robert A. Harrison, José María Gutiérrez, The Neutralization Efficacy Of Expired Polyvalent Antivenoms: An Alternative Option, Toxicon. 2019 October; 168: 32–39. doi:10.1016/j.toxicon.2019.06.216
23. Walsh S, et al. Introducing an E-learning Solution for Medical Education in Liberia, Annals of Global Health. 2018; 84(1), pp. 190–197. DOI: https://doi.org/10.29024/aogh.21
24. Reisach U, Weilemann M. Organisational aspects and benchmarking of e-learning initiatives: a case study with South African community health workers. Glob Health Promot. 2016 Jun;23(2):57-66. doi: 10.1177/1757975914567178. Epub 2015 Mar 2. PMID: 25733133.
25. Barteit S, Jahn A, Banda SS, Bärnighausen T, Bowa A, Chileshe G, Guzek D, Jorge MM, Lüders S, Malunga G, Neuhann F. E-Learning for Medical Education in Sub-Saharan Africa and Low-Resource Settings: Viewpoint. J Med Internet Res. 2019 Jan 9;21(1):e12449. doi: 10.2196/12449. PMID: 30626565; PMCID: PMC6329426.
26. JK Chime, P Munyati , M Katepa-Bwalya , M Musumali , B Mweetwa , S Kagulura , JS Kachimba, B Vwalika , J Mufunda, Using E-Learning for Skills Transfer, Motivation and Retention of Health Workers in Zambia, Medical Journal of Zambia, Vol. 43 (2): PP 103 -105 (2016).
27. Frehywot, S., Vovides, Y., Talib, Z. et al. E-learning in medical education in resource constrained low- and middle-income countries. Hum Resour Health 11, 4 (2013). https://doi.org/10.1186/1478-4491-11-4

28. Rohwer A, Motaze NV, Rehfuess E, Young T. E-learning of evidence-based health care (EBHC) in healthcare professionals: a system atic review. Cam pbell Systematic Reviews 20 17:4, DOI: 10 .40 73/ csr.20 17.4

Tables:

Knowledge, Attitude and Practices of Snakes, Snakebites and their Management among Health Workers in Sudan: Need for internet-mediated Knowledge Management System for Continuing Professional Development.

Table 1. Socio-demographic characteristics of the study population (n=394)

Variables	Character	No. (%)
Sex	Male	176 (44.7)
	Female	218 (53.3)
Age	< 40	142 (36)
	≥ 40	252 (64)
Age	29.3 ± 8	
Occupation	Medical officer	94 (23.9)
	House officer	46 (11.7)
	Registrar	79 (20)
	Consultant	26 (6.6)
	Pharmacist	122 (31)

	Nurse	27 (6.8)
Working Sector	Governmental	229 (58.1)
	Private	165 (41.9)
Years of practice	< 5 years	255 (64.7)
	5 – 10 years	89 (22.3)
	>10 years	50 (13)
Attended training on snakebite management	Yes	102 (25.9)
	No	292 (74.1)
Skills in snake bite management	Self-education (internet or textbooks)	109 (28.5)
	Learning from senior colleagues	142 (37)
	Knowledge and skills gained during training	39 (10.2)
	Don't Know	94 (24.3)

Table 2. The study participants knowledge of snakes and snakebites (n=394).

	Yes (%)	No (%)	I don't know (%)
**Snake Knowledge**			
All snakes are poisonous	36 (9.1)	321 (81.5)	37 (9.4)
All snakes are carnivorous	129 (32.7)	156 (39.6)	109 (27.7)
All snakes are venomous	94 (23.9)	255 (64.7)	45 (11.4)
All snake has fangs in front of their mouth	113 (28.7)	164 (41.6)	117 (29.7)
Snakes pick sounds using their ears	71 (18)	173 (43.9)	150 (38.1)
Snakes are important for farmers	213 (54.1)	89 (22.6)	92 (23.3)
Deforestation and urbanisation have increased human-snake interaction	255 (64.7)	57 (14.5)	82 (20.8)
**Snakebites Knowledge**			
Handling a dead snake's head is safe enough	209 (53)	85 (21.6)	100 (25.4)
Marks can always be seen or found on the victim after every snake bite?	161 (40.9)	195 (49.5)	38 (9.6)

Can a person report at the hospital with symptoms of snake bite without actually being bitten by a snake	219 (55.6)	95 (24.1)	80 (20.3)
Sleeping under mosquito nets can prevent snakebites.	122 (31)	223 (56.6)	49 (12.4)
A venomous ("poisonous") snake bites always injects venom (poison) into the victim	172 (53.7)	151 (38.3)	71 (18)
Signs and symptoms of snake bites are determined by the type of snake responsible for the bite.	316 (80.2)	32 (8.1)	46 (11.7)
The signs and symptoms of a snakebite depend on the amount of venom injected by the snake.	279 (70.8)	60 (15.2)	55 (14)

Table 3: the study participants knowledge of snakes and snakebites (n =394).

**Variable**	**Categories**	**Frequency (%)**
**Snake Knowledge**		
Animal types of snakes	Mammals	31 (7.9)
	Birds	0 (0)
	**Reptiles**	322 (81.7)
	Not Sure	41 (10.4)
**Snakebites Knowledge**		
Ways to determine snake bitten and venom injected into human	Type of snake	78 (19.8)
	Signs and symptoms	209 (53)
	Victim presenting complaints	55 (14)
	I don't know	52 (13.2)
The common time for snakebite	During the day	31 (7.9)
	During the night	274 (69.5)
	Not sure	65 (22.6)

Table 4: The study participants' practice regarding snakebites (n= 394)

**Variable**		
	**Yes (%)**	**No. (%)**
A tourniquet prevents the spread of poison in the remaining part of the body	247 (26.7)	147 (73.3)
Reassurance of the victim should be practice as 1st protocol for prevention	320 (81.2)	74 (18.8)
Incision at the site of bite helps removing poison	186 (47.2)	208 (52.8)
If the bite site is leg, elevating it will reduce poison spread	112 (28.4)	282 (71.6)
Bringing snake to treating physician increases chances of survival by correct identification	297 (75.4)	97 (24.6)

Table 5: The study participants attitude regarding snakebites (n=394)

**Variable**		
	**Yes (%)**	**No (%)**
Residing in cities is a protective factor from snake bite	297 (75.4)	97 (24.6)
Snakebite is an outcome of revenge inspired by past incidents	106 (26.9)	288 (73.1)

Table 6: Management of snakebites (n=394)

Variable	Response	No. (%)
Management of snake bites	Very confident	55 (14)
	Confident	106 (26.9)
	Fairly confident	132 (33.5)
	Not confident	101 (25.6)
Have you ever managed, supervised or nursed a snakebite patient before?	Yes	203 (51.5)
	No	191 (48.5)
Average of snakebites reports to participants health facilities	One person every day	08 (2)
	1 to 5 persons a week	76 (19.3)
	More than five persons a week	22 (5.6)

	1 to 5 persons every month	46 (11.7)
	More than five persons every month	06 (1.5)
	One person every three months	11 (2.8)
	More than five persons every three months	07 (1.8)
	1 to 5 persons in the year	28 (7.1)
	More than five persons in the year	05 (1.3)
	Not Sure	185 (46.9)
The health facility has a protocol for the management of snakebite	Yes	161 (40.9)
	No	144 (36.5)
	I don't know	89 (22.6)
The health facility has what it takes to effectively manage snake bites	Yes	179 (45.4)
	No	215 (54.6)

Table 7: The study participants knowledge of snakebites management (n=394)

Statement	Response	No. (%)
How do you triage (prioritise) someone with a snakebite in your facility?	Emergency	265 (67.3)
	Urgent	102 (25.9)
	Not urgent	07 (1.8)
	I don't know	20 (5)
What do you do when people report to your health facility with snakebite?	Refer immediately	26 (6.6)
	Give first aid treatments and refer	166 (42.1)

	Admit and treat	178 (45.2)
	Call for assistance from another health facility	24 (6.1)
Which of the following tests will you first recommend when someone reports snakebite to determine there was actually an injection of venom into the person (envenoming)?	Full blood count	182 (46.2)
	Grouping and cross matching	65 (16.5)
	20 minutes whole blood count test	61 (15.5)
	Urinalysis for myoglobinuria	86 (21.8)
Antivenoms are the only antidotes in managing snake bites by venomous snakes.	Yes	267 (75.4)
	No	58 (7.1)
	I don't know	69 (17.5)
Antivenoms made anywhere in the world is good for the management of snake bite victims	Yes	199 (50.5)
	No	83 (21.1)
	I don't know	112 (28.4)
Should antivenoms be given to all patients bitten by snakes?	Yes	191 (48.4)
	No	161 (40.9)
	I don't know	42 (10.7)
When an anti-snake venom is injected into the muscle (intramuscular), it is as effective as when injected into the veins (intravenous)	Yes	57 (14.5)
	No	205 (52)
	I don't know	132 (33.5)
Anti-snake venoms remain useful for months or even years after stated expiry dates	Yes	45 (11.4)
	No	152 (38.6)
	I don't know	197 (50)
In using anti-snake venoms, it is better to give low doses repeated over several days than high initial doses.	Yes	132 (33.6)
	No	118 (29.9)
	I don't know	144 (36.5)
