# Supplementary material for: Development and validation of a minimum requirements checklist for snakebite envenoming treatment in the Brazilian Amazonia
Source: PLoS Negl Trop Dis. 2024 Jan 19;18(1):e0011921. doi: 10.1371/journal.pntd.0011921 (PMC10829989; doi:10.1371/journal.pntd.0011921)
Supplement: Attachment — Submitted filename: Response to reviewers.docx [file pntd.0011921.s007.docx]

Dear reviewers,

Thank you kindly for taking the time to review and suggest improvements for our article. We have addressed all the comments raised in an effort to adequately revise this manuscript for publication at PLoS NTD. This letter contains a detailed point-by-point response to each comment.

**Reviewer 2: The methods used are mostly clearly described. However, I don't understand the differentiation of Community Health Centers (CHCs) into Type 1, 2, and 3 in Table 2. Type 1 seems to be a CHC for basic primary health care, but Type 2 and 3 seem to meet the requirements of a hospital. In the discussion section it is written once that Type 3 CHC are in fact hospitals. If a CHC is capable to treat all complications of snakebite envenoming in the Amazon region (Type 3 CHC), which would include respirator treatment, dialysis for Acute Kidney Injury (AKI), blood transfusion, surgical interventions for local cytotoxic venom effects etc., then it is already a referral and even tertiary hospital. This needs clarification.**

Thank you for requesting clarification. We will revise the terminology used here, because you are correct, a Type 3 community health center is in fact a hospital. Type 2 community health centers are an intermediate level of care with additional equipment/supplies, larger infrastructure, and specific training in emergency response than a Type 1 community health center. To compare to the United States, Type 1s are similar to a stationary ambulance (if that), Type 2s are urgent care or small emergency departments, and Type 3s are hospitals or trauma centers.

For snakebite envenoming patients, a Type 1 community health center would function as basic first aid and antivenom administration, as they are closest to where snakebites occur, and we know time is a critical factor to positive health outcomes. After antivenom, if feasible, patients would then be transferred to Type 2 community health centers for observation of potential adverse reactions, clinical management, and follow- up care. Type 3 community health centers (i.e., hospitals) would only be involved in the continuum of care if the patient is severe and referred by a Type 2 or directly by a Type 1.

To make these distinctions clearer in the article, we changed the overall terminology from “community health centers” to “health units” and added additional detail in Table 2 describing each type of health unit.

**Reviewer #2: For the checklist of required equipment, I would add a device (monitor) for continuous ECG and heart rate monitoring, blood pressure and oxygen saturation. This is at least desirable for Type 1 CHCs and essential for Type 2 and 3 CHC (in fact hospitals). This facilitates monitoring of the patients during antivenom administration, which is most important.**

Thank you for your input. In this low-resource setting, cardiac monitors were defined as devices able to continuously measure heart rate, blood pressure, and oxygen saturation. Type 3 units (hospitals) have ECG leads, but Type 2 and Type 1 community health centers do not. Judges considered these cardiac monitors essential for Type 2 and Type 3 health units, as you suggested, where patients are observed during or following antivenom administration (i.e., administered at a Type 1). In Type 1 centers, judges considered cardiac monitors desirable rather than essential because other monitoring devices—sphygmomanometer, pulse oximeter—were deemed essential and can measure these vital signs.

**Reviewer #2: In the Amazon region, Bothrops and Lachesis species are the most important snake species causing >90% of envenoming. They cause cytotoxic and hemotoxic symptoms and for the indication of antivenom administration need a minimum of lab results (blood cell count and 20 minute whole blood clotting test). I don't see any equipment for the possibility to perform these tests, neither in Type 1 nor in type 2 and 3 CHC.**

Thank you for pointing this out. The checklist was developed under the assumption that the test(s) were conducted and there is indication for antivenom; from that point, what are the minimum requirements for administration and clinical management? But, we include antivenom storage requirements in the checklist, and should also have included the indication test(s), as you suggested.

A culturally-relevant clinical practice guideline for antivenom decision-making and administration has been previously developed for this low-resource setting (Rocha et al. 2022). This guide outlines the blood clotting test as the minimum. The supplies required for a clotting test include a plastic syringe, glass tubes, water bath, thermometer, and a chronometer. The checklist you reviewed did not designate glass tubes as essential in Type 1 units, nor a water bath as essential in Type 1 and Type 2 units. We updated these items as essential in all units and explained the rationale in the Results section under the Round 1 subheading.

Regarding labs, the guideline requests blood cell count, urea and creatinine, creatine kinase, urinalysis, and C-reactive protein, but only if feasible. Labs are feasible in Type 2 and Type 3 units, but not Type 1, and this is reflected in the essential versus desirable checklist items.

**Reviewer #2: Decentralized supply of antivenom is an important step forward to improve management of snakebite envenoming, particularly in regions where transport to the next hospital is difficult and time consuming. The authors suggest a checklist to ensure safe administration of antivenom. However, in the discussion section, the importance of well trained health care personnel falls a bit short. Beside equipment, knowledge and experience of health care staff is crucial to manage snakebite victims and avoid death due to adverse reactions during antivenom administration. This cannot be achieved by short training courses, but needs practical experience. A consultation service provided by experienced clinicians would help to support staff in CHCs. Snake identification, dealing with initial life-threatening events (hypovolemic shock, respiratory failure), indication for antivenom, and treatment of side effects needs to be addressed. Health care staff in CHC usually have no experience at all in management of snakebite envenoming and need to have a contact to ask questions and discuss a case in real time. The importance of a consultation service should be mentioned in the discussion part. It will help to improve knowledge and experience of those in charge at CHCs. Does a Poison Control Center exist in Manaus or elsewhere in Brazil? This would be the institution to be contacted and give advice. This is the case, for example, in Thailand where the Poison Control Center in Bangkok can be contacted and asked for advice. At the same time, data on snakebites in the region can be collected. The administration of antivenom has a life-threatening side effect (anaphylactic shock) and there is concern that snakebite victims may die from antivenom side effects. We would do more harm than good. The possibility for health care staff in CHC's to contact experts is therefore absolutely mandatory before a decision to give antivenom is made and to deal with side effects. The staff in CHC should not be left alone with these decisions!**

We agree with your thoughts here. In addition to the minimum Equipment, Supplies, and Medicines, there is a dedicated Human Resources minimum to emphasize the significance of health professionals. The expert judges determined which types of personnel were required and whether additional training was necessary (yes) for antivenom administration and clinical management.

A previous study interviewed health professionals from the Brazilian Amazonia, who described that most of their knowledge comes from more senior professionals in their health unit and direct experience, as you emphasized, because snakebites are frequent in this setting (Murta et al. 2023). Almost all professionals we train have already treated a snakebite. The missing piece is this more formal training and support, such as consultation with experts, also as you suggested. Our team has been conducting trainings with health professionals as funding allows, and established both an online group and an SMS-based care coordination messaging system to connect professionals with each other as well as experts at our institute/hospital, the Foundation for Tropical Medicine in Manaus.

The online group includes doctors, pharmacists, nurses, and even biologists to support these health professionals real-time in the identification and management of snakebite envenoming, from determining the snake species to medication administration. The SMS-based messaging system connects professionals with an expert at our institute/hospital to answer any questions, also in real-time. In addition, health professionals in Type 1 and Type 2 units often communicate with each other via radio or telephone, and engage senior staff to support and advise in snakebite envenoming cases.

We agree that the above is not clear in the manuscript. Our team discussed and added a paragraph emphasizing this care coordination, training, and support for CHC health professionals in the Discussion section under the Feasibility subheading.

To switch gears, regarding your comment on adverse effects associated with antivenom, it is important to note the improvement in antivenom manufacturing over recent decades. There has been a substantial reduction in the frequency and severity of adverse reactions. Currently, it is estimated that 10% of patients might experience some type of adverse reaction to antivenom, usually a low-risk event such as mild skin reaction. This can be treated effectively in a primary health unit, like Type 1 centers, with antihistamines. In previous studies conducted by our research group, which involved monitoring more than 1,000 patients who received antivenom, there were no cases of severe anaphylactic reactions.

However, though rare, a severe adverse reaction could happen, and our team seeks to minimize this risk through the instrument developed and validated in the article. Type 2 and Type 3 health units are capable of treating adverse reactions, and all patients would either be treated at these units or referred there after antivenom administration at a Type 1 unit.

**Reviewer #2: In the discussion section, there is one sentence that I don't understand: "It is worth noting that whether items required for treating reactions were essential to Type 1 CHCs was the largest obstacle to reaching consensus amongst the expert judges. The ultimate decision to not include these items was only accepted given the existing referral pathway from CHCs to hospitals." Actually the CHCs Type 1 are the health facilities where AV is supposed to be used and then all items should be included to treat adverse reactions. Adrenaline, the essential drug to treat anaphylaxis is included, and antihistamines too. Which items were not included?**

Yes, Type 1 CHCs are the health units where antivenom should be used, but currently, antivenom is only administered in Type 3 units (hospitals) due to the distribution structure. This article challenges the system by defining the minimum requirements for antivenom and seeking to accredit Type 1 CHCs. It was difficult for the expert judges, especially those without rural and remote experience in lower resource units like Type 1 and Type 2, to exclude items always available in hospitals and used in rare, severe cases.

Adrenaline and antihistamines were determined essential, as you pointed out; the items not included were related to intensive care, such as a cardiac monitor (discussed in more detail in a previous response above) and more invasive catheters and scalpels.

**Reviewer #3: The Introduction should provide some orientation for the international readers on the epidemiology of snakebite, available antivenoms and severe acute adverse reaction to antivenom in the Brazilian Amazon. In particular, briefly describing (at least by a diagram) the network of healthcare facilities of different levels that involve in snakebite management, their staff involved in managing patients (e.g. whether all these centers are manned by qualified medical doctors and, nurses with emergency care training) would be important in understanding the context.**

Thank you for this suggestion. We agree and added a sentence at the beginning: “In the Brazilian Amazon, the incidence of snakebite envenoming (SBE), though underestimated, is roughly 30,000 cases per annum.” We also created a figure depicting the current care pathways for patients in the Brazilian Amazon versus pathways in the proposed decentralization strategy. Please see the figure and legend below. We hope this clarifies the context.

Figure 1. Snakebite envenoming care continuum in the Brazilian Amazon. A) Care pathways for snakebite patients under the current antivenom distribution structure. B) Care pathways under the proposed antivenom decentralization to community health centers (CHCs).

**Reviewer #3: In the abstract and introduction, the first sentence; “Currently, antivenoms are the only evidence-based, safe, and effective treatment for snakebite envenoming.” This is quite an overstatement. As recent systematic reviews revealed there are no randomized placebo-controlled trials that have shown any effectiveness of antivenoms for the main systemic effects of envenoming such as coagulopathy, neurotoxicity or acute kidney injury. This means there is no highest-quality evidence for the effectiveness of antivenom. However, this doesn't mean that antivenoms are not working at all. Studies that showed the effectiveness of antivenom without a patient group that did not receive antivenom are very few. In addition, antivenoms used in some regions of the world are associated with very high adverse reaction rates (E.g. Indian polyvalent antivenom). A statement like “Currently, antivenoms are the only specific treatment available for snakebite envenoming” would be more realistic.**

Suggestion was accepted accordingly.

We are grateful for the valuable contributions made and are available for any additional suggestions or questions.

Regards,

Wuelton Monteiro
